# Isolated Au Atom Anchored on Porous Boron Nitride as a Promising Electrocatalyst for Oxygen Reduction Reaction (ORR): A DFT Study

**DOI:** 10.3389/fchem.2019.00674

**Published:** 2019-10-17

**Authors:** Qiaoling Li, Tianran Zhang, Xiaofei Yu, Xiaoyu Wu, Xinghua Zhang, Zunming Lu, Xiaojing Yang, Yang Huang, Lanlan Li

**Affiliations:** ^1^Key Lab for Micro- and Nano-Scale Boron Nitride Materials in Hebei Province, School of Materials Science and Engineering, Hebei University of Technology, Tianjin, China; ^2^Department of Chemical and Biomolecular Engineering, National University of Singapore, Singapore, Singapore

**Keywords:** porous boron nitride (p-BN), isolated Au atom, catalysis, oxygen reduction reaction (ORR), density functional theory (DFT)

## Abstract

The development of efficient, stable, and low-cost catalytic material for the oxygen reduction reaction (ORR) is currently highly desirable but challenging. In this work, based on first-principles calculation, the stabilities, catalytic activities and catalytic mechanisms of isolated Au atom supported on defective porous BN (p-BN) have been studied in detail. The results reveal that the defective p-BN anchor Au atom strongly to ensure the stability of Au/p-BN. Based on frontier molecular orbital and charge-density analysis, isolated Au atom supported on porous BN with V_N_ defect (Au/p-BN-V_N_) is an effective ORR catalyst. Especially, the low barriers of the formation (0.38 eV) and dissociation (0.31 eV) of ^*^OOH and the instability of H_2_O_2_ on Au/p-BN-V_N_ catalyst suggest that ORR proceeds via 4-electron pathway. Along the favorable pathway, the reduction of O_2_ to ^*^OOH is the rate-limiting step with the largest activation barrier of 0.38 eV and the maximum free energy change is 1.88 eV. Our results provide a useful guidance for the design and fabrication of new Au-base catalyst with high-efficiency and are beneficial for the developing of novel isolated metal atom catalysts for ORR.

## Introduction

The large-scale commercial applications of electrochemical devices such as polymer electrolyte membrane fuel cells (PEMFCs) are significantly obstructed by sluggish kinetic cathode oxygen reduction reaction (ORR) (Debe, 2012; Sharma and Pollet, 2012; Chen et al., 2019b). Pt-based materials are the best choice for current fuel cell cathodic catalysts. Nevertheless, Pt-based catalysts also suffer from many problems such as high cost, less abundance, the poor tolerance to CO poison and poor stability in an electrochemical environment (Nie et al., 2015). Therefore, great efforts have been devoted to developing novel electrocatalysts for ORR with high efficiency and long-term stability based on low cost and abundant materials.

Au has attracted attention as a promising Pt-alternative catalyst due to its extremely similar crystal structure, low cost, and more abundant than Pt. However, the ORR activity of traditional Au-based catalysts is much lower than that of commercial Pt/C. To date, there are several strategies, such as controlling the shape or morphology of Au nanocrystals (Deng et al., 2015; Alba-Molina et al., 2019), alloying Au with foreign metals (Kodama et al., 2016; Xue et al., 2018), or depositing Au nanoparticles on functional substrate supports (Uosaki et al., 2014; Nie et al., 2015; Kobayashi et al., 2016; Ostojic et al., 2018; Peng et al., 2019). Unfortunately, the catalytic activity of these Au nanoparticles catalysts generally has strongly particle size/shape–dependented and the overall efficiency has been rather low because only the local interface atoms are active for catalysis. Deeply, the fully filled d shell of Au (5 d^10^) is energetically stable, which limits the adsorption and activation of O_2_ and ORR intermediates (Nørskov et al., 2004; Bhatt et al., 2017). Therefore, on the Au-based catalyst, ORR mainly produces H_2_O_2_ via 2-electron process. A rationally design to decrease Au usage and alter Au intrinsic chemical inertness is crucial in enhancing its ORR catalytic activity. Considering the noble nature of Au, downsizing the nanometer-scale to single-atoms could effectively reduce the use of precious metal and maximize atom utilization efficiency. However, up to now, it is not clear whether single-atom Au catalyst can promote ORR process.

As a kind of high-efficiency catalyst, single-atom catalysts (SACs) exhibit extraordinary catalytic activity and selectivity toward various reactions (Bayatsarmadi et al., 2017; Peng et al., 2018; Xiao et al., 2018). Generally, an appropriate support plays a crucial role in realizing isolated metal atom dispersion and immobilize metal atom (Du et al., 2019). Moreover, the strong interaction between the metal and support can modulate the electronic structure of metal catalyst and further improve the catalytic performance. As a catalytic support, BN nanosheet, a structural analog of grapheme, featured with a local polarity B-N bond, has attracted considerable attention due to its excellent acid-base resistance, extraordinary oxidation resistance, and thermal stability (Lin, 2002; Pakdel et al., 2014; Sun et al., 2016; Tran-Thuy et al., 2017). Uosaki et al. demonstrated the possibility to functionalize inert Au substrate to become ORR catalysts by BN modified, reducing the overpotential by ca. 0.27 V (Uosaki et al., 2014). Later, Elumalai and coworkers further reduced the overpotential ca. 50 mV by boron nitride nanosheet (BNNS) supported gold nanoparticles (Elumalai et al., 2016; Lyalin et al., 2017). In addition, BNNS with vacancy defect has been proved to be excellent support for SACs. Feng et al. theoretically predicted that Fe-embedded BNNS with N vacancy defect exhibits a good catalytic activity for ORR as its strong hybridization between the Fe atom and the sp^2^ dangling bonds of nitrogen atoms (Feng et al., 2015). In another study, the catalytic activity of Cu, Co, Au, Pt et al. transition metal atoms doped h-BN nanosheets were also proposed as viable catalysts for CO oxidation (Lin et al., 2013; Deng et al., 2018, 2019).

In this work, a systematic density functional theory (DFT) is performed to explore the potential application of atomic Au anchored on defective porous BN (Au/p-BN) for ORR activity and the ORR mechanism were investigated in detail. Our calculation results revealed that p-BN with vacancy defect (both V_B_ and V_N_) can efficiently anchor Au atom and modify the electronic structure of Au. Moreover, Au/p-BN-V_N_ is a promising catalyst for ORR, because O_2_ is activated effectively on Au/p-BN-V_N_ and reduced to H_2_O via desirable 4-electron process. These results are beneficial to develop new Au-base catalyst with high-efficiency and design novel single-atom catalysts for ORR.

## Computational Models

All the calculations were performed at a spin-polarized density functional theory (DFT) level as implemented in the DMol3 code (Delley, 1990, 2000). Functional of generalized gradient approximation (GGA) with the Perdew, Burke and Ernzerhof (PBE) was used to describe exchange-correlation potential (Perdew et al., 1996). All Electron Relativistic was used for core treatment and the double numerical plus polarization (DNP) was chosen as the atomic orbital basis set (Koelling and Harmon, 1977). The real-space global orbital cutoff radius of 4.6 Å was adopted to ensure high-quality results. The self-consistent field (SCF) procedure was used with a convergence threshold of 10^−6^ au in the energy and electron density (Pulay, 1982). The Brillouin zone was sampled with a 5 × 5 × 1 k-points grid generated automatically by the Monkhorst–Pack method for geometric optimization, while a 9 × 9 × 1 k-points grid was used for electronic structures computations. A conductor-like screening model (COSMO) was used to simulate a H_2_O solvent environment. The dielectric constant was set as 78.54 for H_2_O solvent (Feng et al., 2015; Wang et al., 2015).

As shown in Figure S1, a 3 × 3 × 2 dimension supercell of h-BN was used to construct potential p-BN by introducing vacancies as our previously reported (Li et al., 2016, 2018). The periodic images were separated by a vacuum space of 18 Å in the z direction. The geometry optimizations with the convergence tolerances of energy, maximum force, and displacement on each atom were set to 1.0 × 10^−6^ Ha, 0.001 Ha/Å, and 0.005 Å, respectively. The full optimized unit cell of p-BN contains a central porous ring of 12 atoms. A 2 × 2 × 1 p-BN supercell with 48 B and 48 N atoms was adopted to prevent interactions in periodic images in our study and the lattice parameter was calculated to be 13.65 Å. The interlayer spacing of p-BN was calculated to be 3.821 Å. In this theoretical level, results are in good consistent with those of theorical (Tang et al., 2014) and experimental (Weng et al., 2013; Ye et al., 2016; Tian et al., 2018) studies.

The binding energy (*E*_bind_) of isolated metal atom on p-BN was calculated as:

(1) $$\begin{array}{l} {\;E}_{\text{bind }}=E_{\text{metal}}+E_{\text{p}-\text{BN}}{-E}_{\text{metal}/\text{p}-\text{BN}} \end{array}$$

where *E*_metal_, *E*_p−BN_, and *E*_metal/p−BN_ is the total energy of the isolated metal atom, the p-BN substrate and metal atom/p-BN, respectively.

For the adsorption energy (*E*_ads_), it was defined by:

(2) $$\begin{array}{l} {\;E}_{\text{ads}}=E_{\text{adsorbate}}+E_{\text{substrate }}{-\;E}_{\text{adsorbate}/\text{substrate }} \end{array}$$

where *E*_adsorbate_, *E*_substrate_, and *E*_adsorbate/substrate_, denote the total energy of the free ORR species, substrate and adsorbed species on substrates, respectively. By this definition, positive *E*_ads_ indicates an energetically favorable exothermic process.

The overall binding strength of Au atom in unsupported Au_n_ clusters is characterized by the cohesive energy (*E*_cov_), which is defined by:

(3) $$\begin{array}{l} {\;E}_{\text{cov}}=\left[nE\left(Au\right)-E\left(Au_{\text{n}}\right)\right]/n \end{array}$$

where *E*(Au) and *E*(Au_n_) represent the calculated energies of an Au atom and the unsupported Au_n_ cluster, respectively and n is the number of atom in the cluster.

For Au_n_ clusters supported on p-BN-V_N_, *E*_cov_ can be computed as the following:

(4) $$\begin{array}{l} \;E_{\text{cov}}=[nE\left(Au\right)+E(p-BN-V_{\text{N}})\\ \;-E(Au_{\text{n}}/p-BN-V_{\text{N}})]/n \end{array}$$

where *E*(p-BN-V_N_) and *E*(Au_n_/p-BN-V_N_) represent the calculated energies of p-BN-V_N_ support and the Au_n_/p-BN-V_N_, respectively.

The transition states for ORR elemental steps were obtained by complete LST/QST tools in DMol3 code (Govind et al., 2003), and frequency calculations were performed to confirm the obtained transition states. Free energies of the ORR intermediates in electrochemical reaction pathways were calculated based on the computational hydrogen electrode (CHE) model proposed by Peterson et al. (2010). The Gibbs free energy (Δ*G*) change of each elementary step in the ORR was determined by:

(5) $$\begin{array}{l} \Delta G=\Delta E+\Delta E_{\text{ZPE}}-T\Delta S \end{array}$$

(6) $$\begin{array}{l} E_{\text{ZPE}}=\;\frac{1}{2}\sum \hbar \nu \end{array}$$

The reaction energy (Δ*E*) is the total energy change directly obtained by DFT calculation. Δ*E*_ZPE_ and Δ*S* are the zero–point energy difference and the entropy change between the products and reactants, respectively. T is the system temperature in this work (T = 298.15 K). For each system, its *E*_ZPE_ can be implemented by summing vibrational frequencies over all normal modes. The entropies of the free molecules (O_2_, H_2_, H_2_O, H_2_O_2_) were taken from the NIST database [Computational Chemistry Comparison and Benchmark Database.http://cccbdb.nist.gov/].

## Results and Discussion

### Geometry and Stability of Au/p-BN

**Figure 1:**
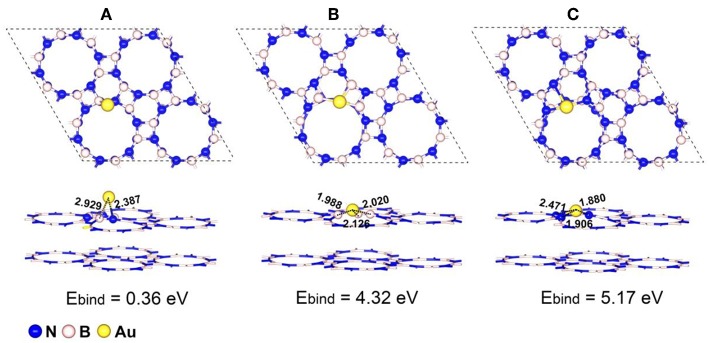
Optimized geometric configurations with the corresponding binding energy (*E*_bind_/eV) of single atom Au anchored on a perfect p-BN **(A)**, p-BN with V_N_ defect **(B)**, and p-BN with V_B_ defect **(C)**, respectively. The bond lengths are given in Å.

We firstly examined all the possible anchored sites for single Au atom on both perfect p-BN and defective p-BN including single nitrogen (V_N_) and boron (V_B_) vacancies and the most stable configurations for Au atom anchored on p-BN are shown in Figure 1. As shown in Figure 1A, the Au atom locates on the bridge site over boron-nitride bond for perfect p-BN and the bond length for Au-B and Au-N are calculated to be 2.929 and 2.387 Å respectively, which are much shorter than the van der Waals distance of 3.790 and 3.210 Å. With the introduction of V_N_ (V_B_) defects (Au/p-BN-V_N_, Au/p-BN-V_B_), Au atom locates at the center of the defect, forming three non-equivalent Au-B (Au-N) bonds with the distance of 2.126, 1.988, and 2.020 Å (1.906, 2.471, and 1.880 Å), respectively (Figures 1B,C). Compared with Au/p-BN, the shorter bond lengths reveal the stronger interaction between the Au atom and the support. As radius of the Au atom is larger than that of the missing N/B atom, the anchored Au atom protrude from the basal plane of p-BN surface by about 0.631 and 0.544 Å for Au/p-BN-V_N_ and Au/p-BN-V_B_ respectively.

For an effective SACs, the strong binding strength between metal atoms and support is an ultimate prerequisite to protect metal atom from its aggregation and thus keep its good stability for long-term uses (Yang et al., 2013). In order to evaluate the stability of isolated Au atom on p-BN, the binding energies (*E*_bind_ /eV) of Au atom on both perfect and defective p-BN were calculated. For perfect p-BN, the *E*_bind_ was calculated to be 0.36 eV. With the introduction of vacancies, the *E*_bind_ increased to be 4.32 and 5.17 eV for V_N_ and V_B_, respectively, which is larger than corresponding cohesive energy (*E*_cov_ = 3.76 eV, Figure S2) evidently, indicating that the defect structures are crucial in producing stable Au/p-BN catalyst. It is noteworthy that the *E*_bind_ for Au atom on defective p-BN is much larger than that for Au on other substrates reported such as on defective h-BN (3.48 eV for V_N_ and 3.72 eV for V_B_, PBE) (Gao et al., 2012), on CeO_2_ (2.11 eV, PBE+U) (Song and Hensen, 2013), and on TiO_2_ with oxygen vacancy (1.28 eV, PBE) (Wan et al., 2018) etc. The larger *E*_bind_ of Au atoms on defective p-BN also makes the diffusion of Au atom to its neighboring site is considerably difficult in terms of the large endothermicity and high energy barrier, thus vigorously excluding the clustering of anchored Au atom on this support. We further considered the migration of Au atom on V_N_ by the diffusion energy diagrams (Figure S3) (Chen et al., 2019a), which is relevant for particle aggregation. Two different pathways for the diffusion of the most stable Au on the V_N_ were considered and the activation energies are calculated to be 3.46 and 2.36 eV, respectively. These large migration activation energies suggest that the Au atom is dynamically stable on the adjacent 3B site on V_N_. Therefore, these results clearly show that the defective p-BN anchor Au atom strongly to ensure the stability of Au/p-BN.

**Figure 2:**
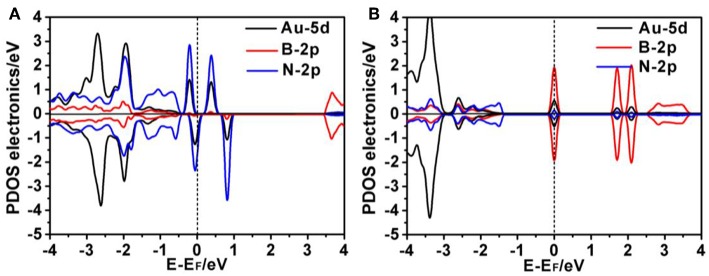
Projected density of states (PDOS) for the Au/p-BN-V_B_ **(A)** and Au/p-BN-V_N_ **(B)**, respectively. The position of the Fermi level was marked in dashed line.

To gain a better understanding of the interaction between the Au atom and defect sites of p-BN, we performed electronic structure analyses by calculating the partial density of states (PDOS), frontier molecular orbital and total charge density and charge-density difference. As shown in Figures 2A,B, the PDOS for Au/p-BN-V_B_ and Au/p-BN-V_N_ demonstrates that the impurity states mainly consist of the 5d orbital of Au atom and 2p orbital of its adjacent N and B atoms, respectively. Meanwhile, the evident resonance between the 5d orbital of the Au atom and the 2p orbital of N/B atoms are observed, revealing a strong interaction between the Au atom and B/N-vacancy. The strongly interaction indicates that Au atom can be favorably supported on the defect of p-BN in thermodynamics. This is due to so-generated defect states in defective p-BN that affected the system's adsorption ability and catalytic activity (Figure S4) (Li et al., 2016, 2018). In particular, it is found that there is one evident half-occupied d state cross the Fermi level for Au/p-BN-V_N_ (Figure 2B). This forceful d state around the Fermi level is generally considered to be an indicator of the high activity, which play a crucial role in catalysis (Deng et al., 2019).

**Figure 3:**
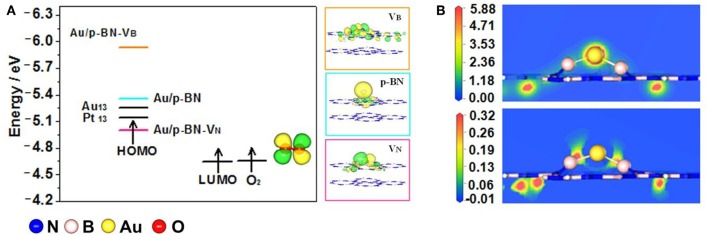
Frontier molecular orbital diagram shows the interaction of acceptor O_2_ with donor catalysts Au/p-BN, Au/p-BN with V_N_, V_B_, Au_13_, and Pt_13_. HOMO shapes of Au supported on Au/p-BN, Au/p-BN with V_N_, V_B_, and LUMO shape of O_2_ **(A)**. Total charge density and Charge-density difference plots of Au anchored on V_N_ **(B)**.

Figure 3A displays the highest occupied molecular orbital (HOMO) energies of the Au/p-BN, Au/p-BN-V_N_, Au/p-BN-V_B_ catalyst and the lowest unoccupied molecular orbital (LUMO) energy of O_2_ molecule. For comparison, pure Au_13_ cluster and Pt_13_ cluster were also calculated and displayed. In the case of the ORR occurring on catalyst, the O_2_ molecule obtains electrons from the catalyst and then is reduced to H_2_O, that is, electrons on the HOMO of catalyst transfer to the LUMO of O_2_ during ORR. From the viewpoint of frontal molecular orbit theory (Zhou, 1993), the smaller the energy difference between the HOMO and the LUMO, the more easily an electron transfer takes place. As shown in Figure 3A, on pure Pt_13_, the energy difference between the HOMO of catalyst and the LUMO of O_2_ is less than that on pure Au_13_, indicating that Pt is a better catalyst than Au for ORR. The HOMO shifts to lower energy after Au has been loaded onto both p-BN and V_B_ support. Consequently, the energy difference between the HOMO of catalyst and the LUMO of O_2_ increased and the electron transfer between Au/p-BN (Au/p-BN-V_B_) and O_2_ is more difficult even than Au_13_ cluster. Hence, neither Au/p-BN nor Au/p-BN-V_B_ is suitable as the catalyst for ORR. Whereas, among all of the modeled catalyst, Au/p-BN-V_N_ catalyst shares the minimized energy difference between the HOMO of catalyst and the LUMO of O_2_ and this energy difference is even less than that of pure Pt_13_. In other words, electron transfer can more smoothly proceed from the HOMO of Au/p-BN-V_N_ to the LUMO of O_2_. These results indicate that the V_N_ modifies the Au/p-BN-V_N_ electron structure such that Au/p-BN-V_N_ may be more beneficial than Pt for ORR. Thus, we would only investigate the ORR activity and mechanism of isolated atomic Au loaded on p-BN with V_N_ defect (Au/p-BN-V_B_) in the following discussion.

To further understand the regulation of Au electronic structure by V_N_ defect, we analyze the total charge density and charge-density difference as shown in Figure 3B. From the contours of charge-density difference, there is obvious charge enrichment in B atoms together with charge depletion in Au atom, indicating electrons are transferred from the adsorbed Au to the defective p-BN. Indeed, the bader charge analysis confirms that 0.595 e were transferred from Au atom to the B atoms around V_N_. This is due to the B atoms around V_N_ have less electrons than the N atoms, indicating the B p orbitals accepting electrons from the Au d orbital (Figure S5).

### The Adsorption of the ORR Intermediates

**Figure 4:**
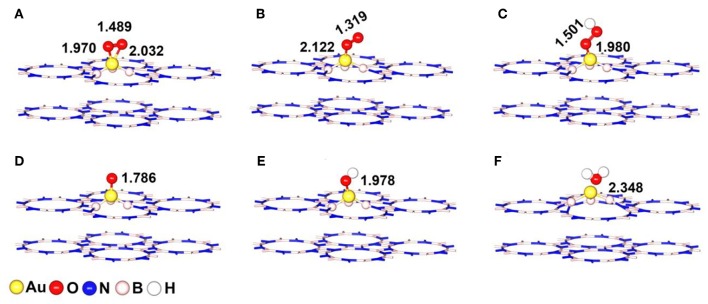
The most stable adsorption configurations of ORR intermediates on Au/p-BN-V_N_ catalyst **(A)** ^*^O_2_-Bridge, **(B)** ^*^O_2_-Pauling, **(C)** ^*^OOH, **(D)** ^*^O, **(E)** ^*^OH, and **(F)** H_2_O. The key bond lengths are given in Å.

In this section, we considered the adsorption of ORR key intermediates (^*^O_2_, ^*^OOH, ^*^O, ^*^OH, and H_2_O) on Au/p-BN-V_N_ to investigate its catalytic activity, where, the ^*^ denotes the adsorption state on the Au/p-BN-V_N_ surface. For each species, different adsorption sites and various adsorption configurations were considered, and the favored configurations are shown in Figure 4.

**Figure 5:**
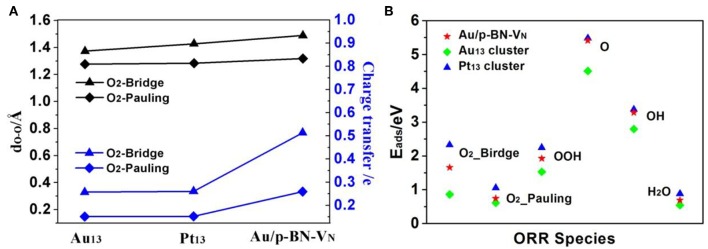
The O-O bond lengths and charge transfer amount of the adsorbed ^*^O_2_ species on Au/p-BN-V_N_, Pt_13_, and Au_13_ catalyst **(A)**. The adsorption energies (*E*_ads_) of the ORR intermediates adsorption on Au/p-BN-V_N_, Pt_13_ and Au_13_ **(B)**.

The chemisorption of molecular O_2_ on catalyst surface is a crucial step for effective ORR as its initial adsorption manner plays an important role in the subsequent reaction pathways. Hence, we discuss the adsorption of O_2_ firstly by considering two initial configurations including a Bridge (side-on) and a Pauling (end-on) with the minimum energy configurations (Figures 4A,B). For Bridge-adsorption, two nonequivalent Au-O bonds are formed with the distances of 1.970 Å and 2.032 Å, respectively. Both are shorter than that of Au-O (2.122 Å) for pauling-adsorption. After adsorption, the O-O bond length of ^*^O_2_ elongates from 1.225 Å to 1.489 Å and 1.319 Å for the Bridge and Pauling configurations, respectively. It is worth noting that the O-O bond length not only longer than that of ^*^O_2_ adsorbed on Au_13_ cluster but also longer that of ^*^O_2_ adsorbed Pt_13_ cluster, as the depiction in Figure 5A. The elongated O-O bond suggests that O_2_ can be more easily activated and dissociated, that is, Au/p-BN-V_N_ catalyst benefits the activation and dissociation of molecular ^*^O_2_ easier. To get more insight into the activation of ^*^O_2_ by Au/p-BN-V_N_, Au_13_ and Pt_13_ catalysts, the Hirshfeld charge (Hirshfeld, 1977) were calculated. The results reveal that there are 0.513 and 0.259 e were transformed from the Au/p-BN-V_N_ to O_2_ molecule for the Bridge and Pauling configurations, respectively, which larger than those from pure Au_13_ (0.257 e and 0.151 e) and Pt_13_ (0.260 e and 0.152 e). Moreover, from contours of the charge-density difference plots (Figure S6), it can be seen that certain amount of charge is transferred from Au atoms around V_N_ vacancy to the O_2_ molecule, concomitant with the formation of the O-Au bond between O_2_ and Au/p-BN-V_N_. The increases in charge transfer lead to the elongation of the O–O bond of ^*^O_2_, subsequently the activation of ^*^O_2_ on Au/p-BN-V_N_. In addition, we further calculated the O_2_ adsorption on isolated Pt and Pd supported on defective p-BN to compare the ORR catalytic activity. The Au/p-BN-V_N_ possess more superior ORR catalytic performance than Pt and Pd atom loaded on p-BN due to the large *E*_ads_ value, O-O bond elongation, and more electron transfer between O_2_ and catalyst (Table S1).

For the OOH, O, OH and H_2_O species, the most favorable adsorption configurations were displayed in Figures 4C–F. After adsorbed on Au/p-BN-V_N_, the O-Au (*d*_O−Au_) bond is formed and the bond length is calculated to be 1.980, 1.786, and 1.978 Å for ^*^OOH, ^*^O, and ^*^OH species, respectively. In the case of ^*^OOH species, the O-OH is elongated from 1.341 Å of the isolated OOH to 1.501 Å, indicating the activation of the O-OH bond. Furthermore, the H_2_O is 2.348 Å far away from the catalyst surface, suggesting weak interaction between H_2_O and Au/p-BN-V_N_ catalyst.

Further, the adsorption energies (*E*_ads_) of ^*^O_2_ and other ORR intermediates including ^*^OOH, ^*^O, ^*^OH and H_2_O on Au/p-BN-V_N_, Au_13_, and Pt_13_ cluster are summarized in Figure 5B. The *E*_ads_ of these species on pure Au_13_ cluster are all the least, revealing the Au cluster itself is very inert and the adsorption on Au_13_ cluster is unfavorable. The *E*_ads_ of ^*^O_2_ on Au/p-BN-V_N_ was computed to be 1.66 eV and 0.72 eV for the Bridge and Pauling models, respectively, which is larger than that of ^*^O_2_ on Au_13_ cluster (0.86 and 0.62 eV) but smaller than that of ^*^O_2_ on Pt_13_ cluster (2.33 and 1.05 eV). In particular, the *E*_ads_ of ^*^O and ^*^OH species on Au/p-BN-V_N_ is large up to 5.41 and 3.28 eV respectively, comparable to the *E*_ads_ of ^*^O and ^*^OH species on Pt_13_ (5.47 and 3.37 eV). Generally, the adsorption strength of ^*^O and ^*^OH species on transition metal catalyst depends upon the electronic configuration of d-electrons of metal, which is primarily affected by whether the d shell is fully occupied or not. The fully filled d shell of Au is energetically stable, which is likely to have a lower the d-band center (Bhatt et al., 2017). Therefore, pure Au cluster is energetically stable as its fully filled d shell. But for Au/p-BN-V_N_ catalyst, the Au atom with a partially filled d shell has a stronger *E*_ads_ for oxygenated species. The smaller *E*_ads_ illustrate the weak interaction between H_2_O and catalysts, suggesting that the final product H_2_O can speedy drift away from the catalyst surface, avoiding the flooding of the catalyst. In addition, we consider the effects of water environment on the adsorption by using a conductor-like screening model (COSMO) to simulate a H_2_O solvent environment, which is shown to be an effective method to describe the solvation and may exhibit excellent consistency with experiment (Wang et al., 2015). As shown in Table S2, the adsorption energies of ^*^O_2_ species on Au/p-BN-V_N_ are increased slightly by solvation, while few impact on other species. Thus, water slimly stabilizes the adsorbed O_2_ species that exhibits positive effects on the ORR process.

The deterioration of the cathode catalyst can largely be attributed to H_2_O_2_ species, an intermediate by a 2e^−^ transfer reaction of ORR. Here, we examined the adsorption of ^*^H_2_O_2_ molecules on Au/p-BN-V_N_, Au_13_, and Pt_13_ catalysts as shown in Figure S7. The adsorption of ^*^H_2_O_2_ on Au_13_ cluster is stable with a small bond length change on bond length of H_2_O_2_ molecule (*d*_O−O_ = 1.525 Å). When adsorbed on Pt_13_ cluster, the O-O bond length of ^*^H_2_O_2_ elongate from 1.469 Å of isolated H_2_O_2_ molecule to 1.796 Å, suggesting the activation of O-O bond. Notably, the O-O bond length is further elongated to 2.832 Å on the Au/p-BN-V_N_ catalyst, accompanying with the generation of a H_2_O molecule and retention of O species, revealing that ^*^H_2_O_2_ is unstable on the Au/p-BN-V_N_ catalyst and is likely to decompose into H_2_O and O species immediately. Accordingly, ORR process occurs on the Au/p-BN-V_N_ catalyst surface via a direct 4e^−^ pathway. Combined the frontal molecular orbit analysis, the activation of O-O bond and the adsorption of ORR intermediates, we can ascertain that Au/p-BN-V_N_ is beneficial to the catalysis of ORR.

### Mechanism of ORR on Au/p-BN-V_N_ Catalyst

The elementary reactions mechanism for the ORR on Au/p-BN-V_N_ were further investigated, including: ^*^O_2_ dissociation, ^*^OOH formation or dissociation, ^*^H_2_O_2_ formation, ^*^OH formation, and H_2_O formation, as the follows:

(1-a) O∗ 2→ O∗ + O∗

(1-b) O∗ 2+ H++ e-→O∗OH

(2-a) O∗OH+ H++ e-→H2O2 *

(2-b) O∗OH+ H++ e-→H2O+ O∗

(2-c) O∗OH→O∗H +O∗

(3) O∗+ H++ e-→O∗H

(4) O∗H+ H++ e-→H2O

**Figure 6:**
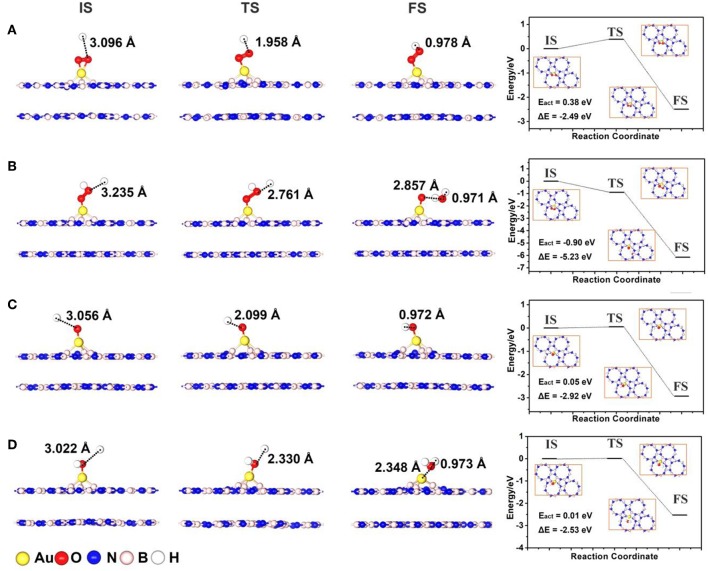
The structures for initial state (IS), transition state (TS), final state (FS), and potential energy profile for ^*^O_2_ + H^+^ + e^−^ → ^*^OOH **(A)**, ^*^OOH + H^+^ + e^−^ → H_2_O + ^*^O **(B)**, ^*^O + H^+^ + e^−^ → ^*^OH **(C)** and ^*^OH + H^+^ + e^−^ → H_2_O **(D)** on a Au atom supported p-BN with V_N_.

As shown in Figure 6, we confirmed the initial states (IS) and final states (FS) of various elementary reactions of the ORR on Au/p-BN-V_N_ according to the most favorable adsorption sites, corresponding transition states (TS) are determined by the complete LST/QST method subsequently. The atomic configurations at various states along ORR reaction paths and corresponding heats of reaction and activation energies are also summarized in Figure 6.

After the ^*^O_2_ adsorption, it can either undertake decomposition to form two adsorbed ^*^O (1-a) or generate ^*^OOH with the capture of one H^+^ and an additional electron (1-b). In pathway (1-a) (Figure S8A), the direct ^*^O_2_ dissociation begins with the minimum energy state of adsorbed ^*^O_2_ with Bridge configuration. The dissociation proceeds with the stretching of O-O bond above Au site, such that each O atom is near a Au-B bridge bond at the transition state. The calculated activation barrier (*E*_act_ = 1.32 eV) for ^*^O_2_ dissociation step on Au/p-BN-V_N_ is similar with that on Au_13_ cluster (*E*_act_ = 1.01 eV, Figure S9A), as well as the reaction energy is less favorable (Δ*E* = 1.14 eV). Obviously, the direct dissociation of ^*^O_2_ on Au/p-BN-V_N_ via pathway (1-a) is not the most favorable way. Contrarily, the activated ^*^O_2_ on Au/p-BN-V_N_ can easily capture one H^+^ and an additional e^−^ to form an ^*^OOH species via the pathway (1-b). Remarkably, this step is exothermic with 2.49 eV and the reaction energy barrier is only 0.38 eV (Figure 6A), indicating the feasibility of ^*^O_2_ protonation process on the Au/p-BN-V_N_ layers.

Next, three possible reaction pathways (2a-c) were also identified, corresponding to the protonation and dissociation process of ^*^OOH on catalyst. Generally, the next reaction may proceed in the following ways: (1) the further hydrogenation of ^*^OOH may result in the formation of H_2_${\text{O}}_{2}^{*}$ (by the pathway 2-a) leading to unfavorable 2-electron process, (2) the ^*^OOH undergoes further protonation reduction to form a H_2_O molecule and one ^*^O species on catalyst surface (by the pathway 2-b), and (3) O-OH dissociate into^*^O and ^*^OH (by the pathway 2-c). However, the 2e^−^ reduction pathway 2-a is impossible as the adsorption of the H_2_O_2_ on Au/p-BN-V_N_ is unstable discussed in the previous section 3.2. Therefore, we only consider the pathway 2-b and 2-c. Amazingly, the protonation process of OOH^*^ on the Au/p-BN-V_N_ catalyst (^*^OOH + H^+^→^*^O + H_2_O) is exothermic by −5.23 eV with no energy barrier at all (Figure 6B). Therefore, once the ^*^OOH species is formed, it can be further hydrogenated by reacting with another H^+^. In contrast, the ^*^OOH are easy to form H_2_O_2_ on the Au_13_ with a low activation energy barrier (*E*_act_ = 0.08 eV) as shown in Figure S9B, which is consist with the reported results (*E*_act_ = 0.07 eV) of Au(111) catalyst (Yang et al., 2017). In addition, the ^*^OOH group also can directly dissociate into ^*^O + ^*^OH via pathway (2-c) on the Au/p-BN-V_N_ catalyst with a small activation energy barrier (*E*_act_ = 0.31 eV) and benefited exothermic reaction energy (−1.95 eV) as shown in Figure S8B. Finally, we also considered the kinetics for the subsequent reductions of ^*^O to ^*^OH and ^*^OH to H_2_O, the activation energy barriers were 0.05 eV and 0.01 eV, respectively (Figures 6C,D). Overall, the ORR on the Au/p-BN-V_N_ is a 4e^−^ transformation pathway, which breaks the convention of a 2e^−^ reduction from O_2_ to H_2_O_2_ on other Au-based catalysts (Uosaki et al., 2014; Yang et al., 2017). Along the favorable pathway, the reduction of O_2_ to ^*^OOH is the rate-limiting step with the largest activation barrier of 0.38 eV on Au/p-BN-V_N_ catalyst. This value of *E*_act_ is smaller than that of the reported single Pt on TiC(111) catalysts (*E*_act_ = 0.51 eV) and pure Pt(111) surface (*E*_act_ = 0.79 eV) (Duan and Wang, 2011; Tak et al., 2018).

**Figure 7:**
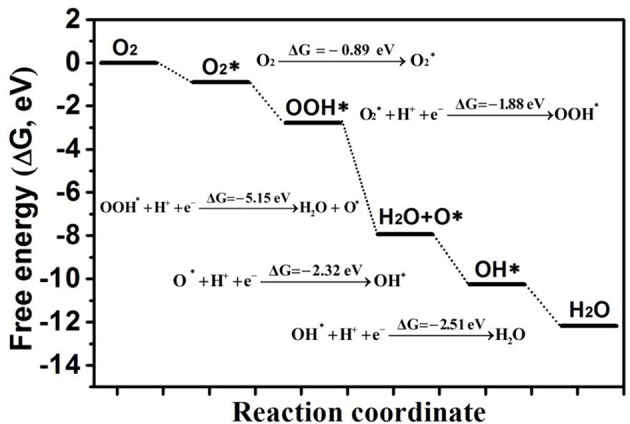
The free energies profile of whole ORR pathways on Au/p-BN-V_N_.

Finally, the free energy change of each ORR step of the most favorable reaction route (as shown in Figure S10) on Au/p-BN-V_N_ was listed in Figure 7. Here, the energy state of the first step is the total energy of both Au/p-BN-V_N_ and O_2_ molecule. Then, the reference energy state of the next reduction steps is the total energy of the products of previous reaction step and H^+^ + e^−^. The ORR proceeds mainly through three forms are characterized as the O_2_ activation, hydrogenation reduction, and O-OH breaking processes. For the first step of the reaction, the adsorption of O_2_, is exothermic and downhill in the free energy profile by 0.89 eV. Then, the adsorbed O_2_ molecule prefers to be hydrogenated to form an OOH molecule and the Gibbs free energy decreases by of 1.88 eV. Furthermore, the formed OOH species is hydrogenated to form a H_2_O and leave one ^*^O on the Au/p-BN-V_N_ site with the Δ*G* of 5.15 eV. Subsequently, the ^*^O atom is converted into OH with the help of H and this process is downhill in the free energy profile by 2.32 eV. Finally, the ^*^OH is further reduced to form another H_2_O molecule with a downhill the free energy profile by 2.51 eV.

## Conclusions

In summary, by density functional theory computations, the stability as well as its catalytic activities for ORR of the single atom Au supported on p-BN layers was systematically investigated. The study demonstrated that Au/p-BN-V_N_ exhibits both high stability and excellent catalytic activities for ORR. Specifically, Au/p-BN-V_N_ has a large *E*_bind_ value (4.32 eV) which is even higher than the *E*_cov_ of bulk Au (3.76 eV) and higher migration barrier (*E*_act_ = 2.36~3.46 eV), which can prevent the aggregation and ensure the stability of Au/p-BN-V_N_ catalyst. It was found that the positive charge density of B3 site among V_N_ defect is beneficial to the electrons transfer from the d shell filled Au atom to B atoms, which is beneficial to break the inert state of Au-d^10^ and contribute to the adsorption and activation of ORR species. Notably, the ^*^H_2_O_2_ is found to be quite unstable on the Au/p-BN-V_N_ catalyst and tend to decompose into H_2_O and ^*^O species immediately. Moreover, the activation energy of each reaction pathway and the free energies profile of whole ORR pathways on Au/p-BN-V_N_ were estimated and analyzed. By comparison of activation energy of each elementary reaction, we find that the reduction of the O_2_ to OOH is the rate-limited step with the largest activation barrier of 0.38 eV. Overall, these results would shed light on the understanding of Au-based catalyst for ORR and motivate the investigations on ORR kinetic behavior of isolated metal atom supported on p-BN catalysts.

## Data Availability Statement

The datasets generated for this study are available on request to the corresponding author.

## Author Contributions

All authors have made a substantial contribution to the work and approved its publication. QL and LL designed the protocol, made calculations, and wrote the paper. TZ made calculations. XYu, XW, XZ, and ZL entered the discussion. XYa, YH, and LL revised the paper.

### Conflict of Interest

The authors declare that the research was conducted in the absence of any commercial or financial relationships that could be construed as a potential conflict of interest.
