# Supplementary material for: Isolated Au Atom Anchored on Porous Boron Nitride as a Promising Electrocatalyst for Oxygen Reduction Reaction (ORR): A DFT Study
Source: Front Chem. 2019 Oct 17;7:674. doi: 10.3389/fchem.2019.00674 (PMC6811612; doi:10.3389/fchem.2019.00674)
Supplement: Supplementary file 1 [file Table_1.docx]

Supplementary Material

**1 Supplementary Figures**


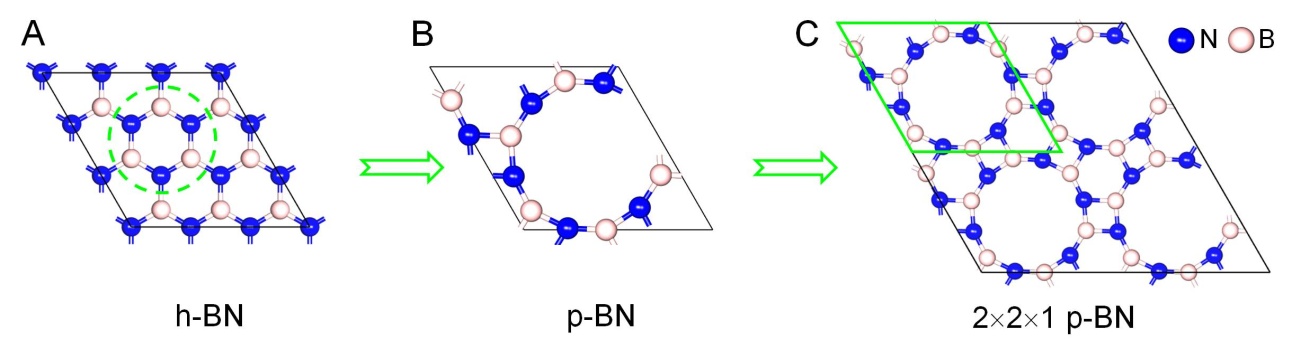


**FIGURE S1**│The geometries of original h-BN **(A)**, optimized primitive cell porous BN (p-BN) **(B)**, and supercell 2×2×1 p-BN **(C)**, respectively.





**FIGURE S2│**The cohesive energies of isolated Au_n_ cluster, Au_n_/p-BN-V_N_ (n=2~13) and Au bulk.


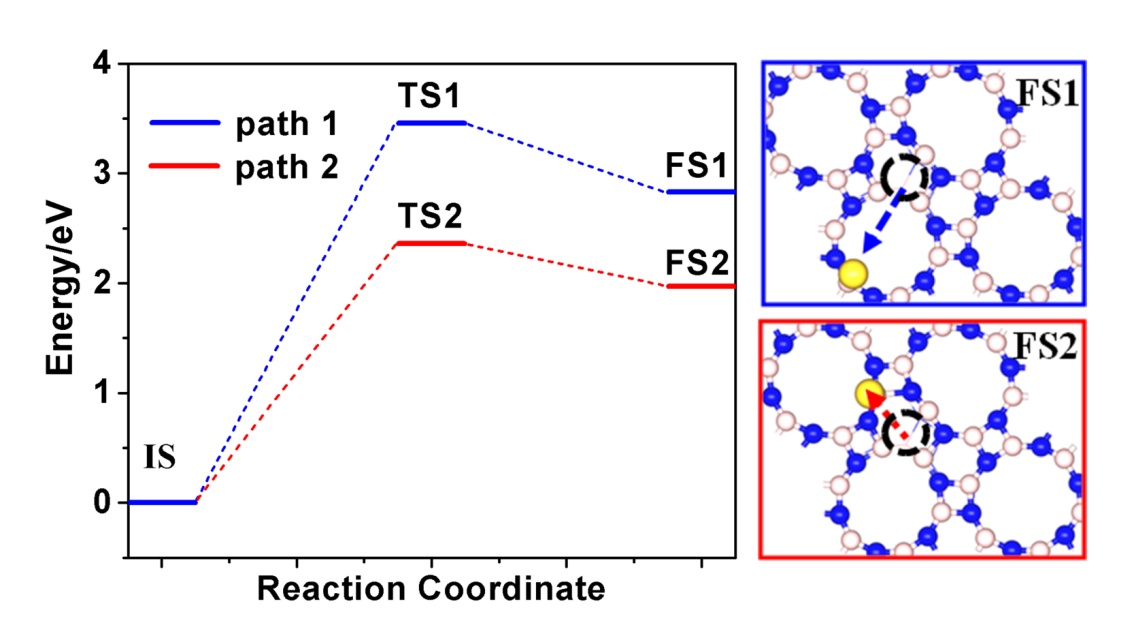


**FIGURE S3│**Potential energy diagrams for the migration of a single Au atom on V_N_ vacancy.


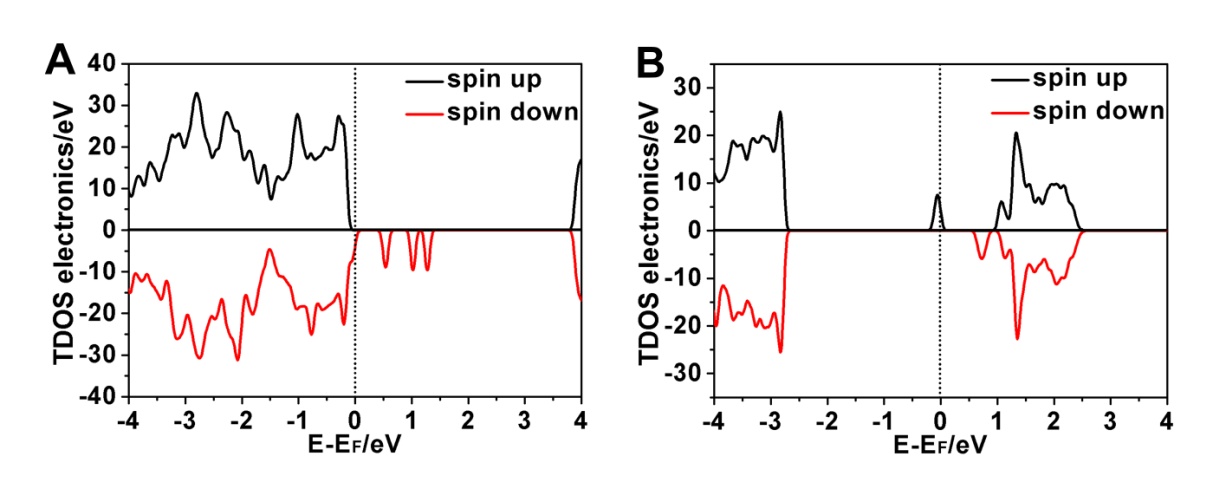


**FIGURE S4│**Total density of states (TDOS) for p-BN with V_B_ defect (A) and p-BN with V_N_ defect (B).

**
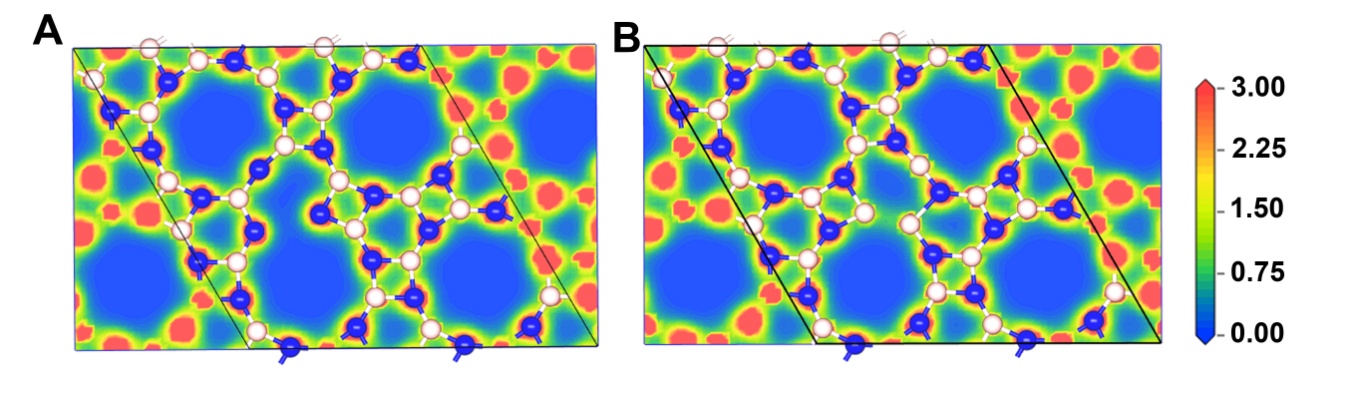
**

**FIGURE S5│**The total charge density of p-BN with V_B_ defect (A) and p-BN with V_N_ defect (B).


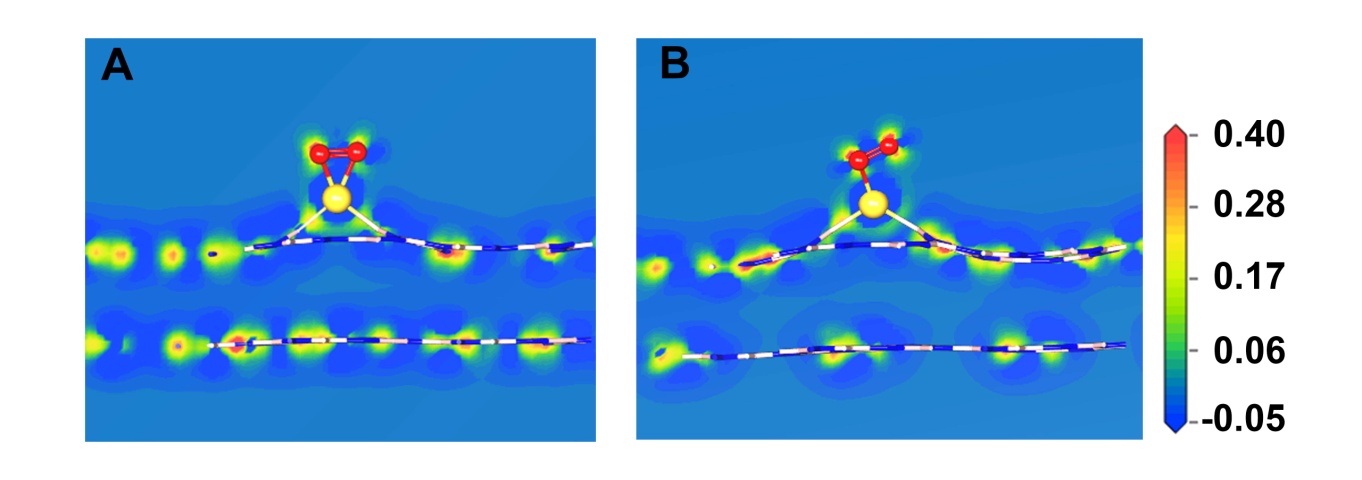


**FIGURE S6│** The charge-density difference plots of O_2_ adsorbed on Au/p-BN-V_N_: **(A)** *O_2_-Bridge, **(B)** *O_2_-Pauling.


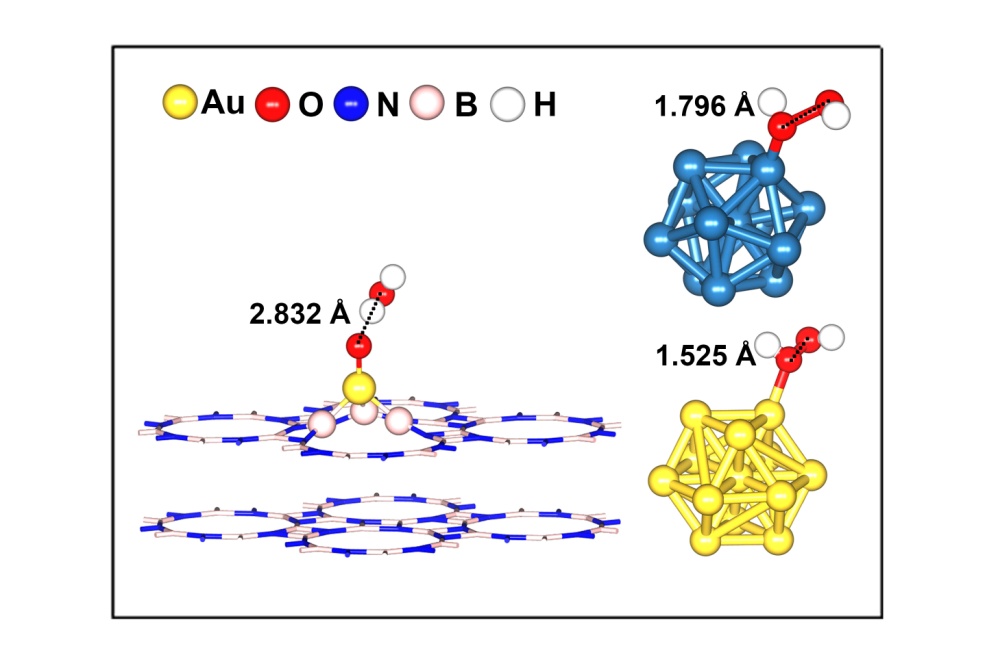


**FIGURE S7**│The geometry structures of H_2_O_2_ adsorption on Au/p-BN-V_N_, Pt_13_ and Au_13_ cluster.


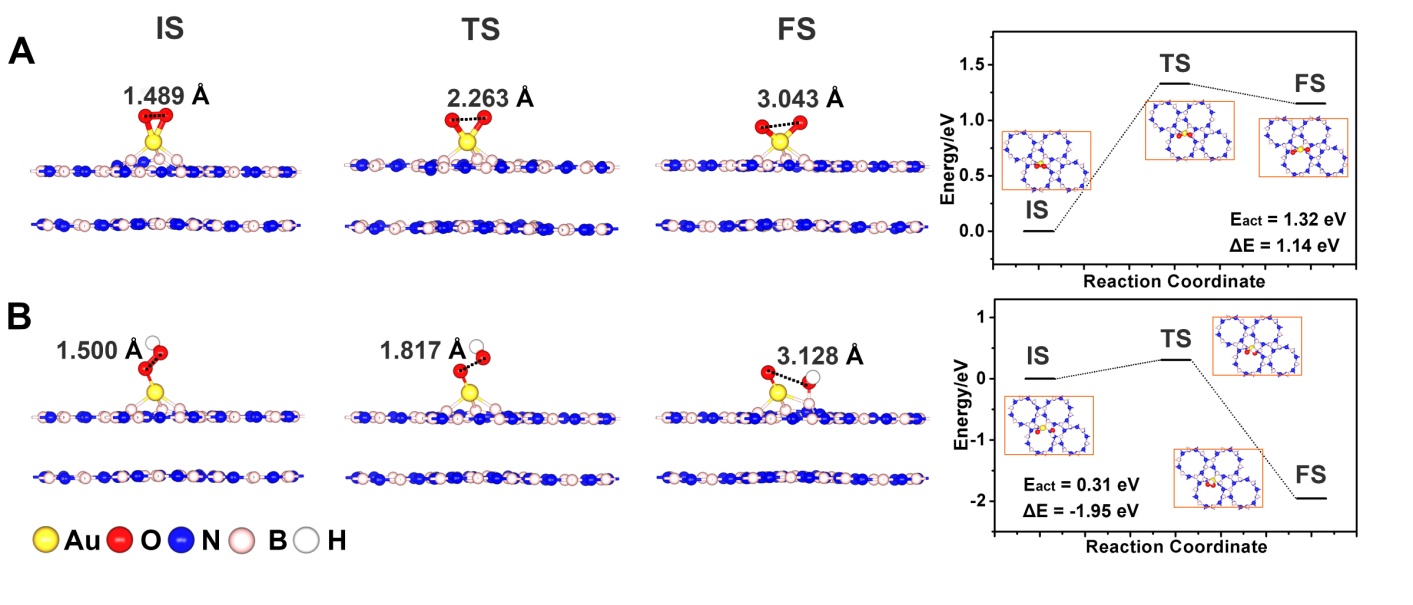


**FIGURE S8**│The structures for initial state (IS), transition state (TS), final state (FS) and potential energy profile for **(A)** *O_2_→ *O + *O, **(B)** *OOH → *OH + *O on a Au atom supported p-BN with V_N_.


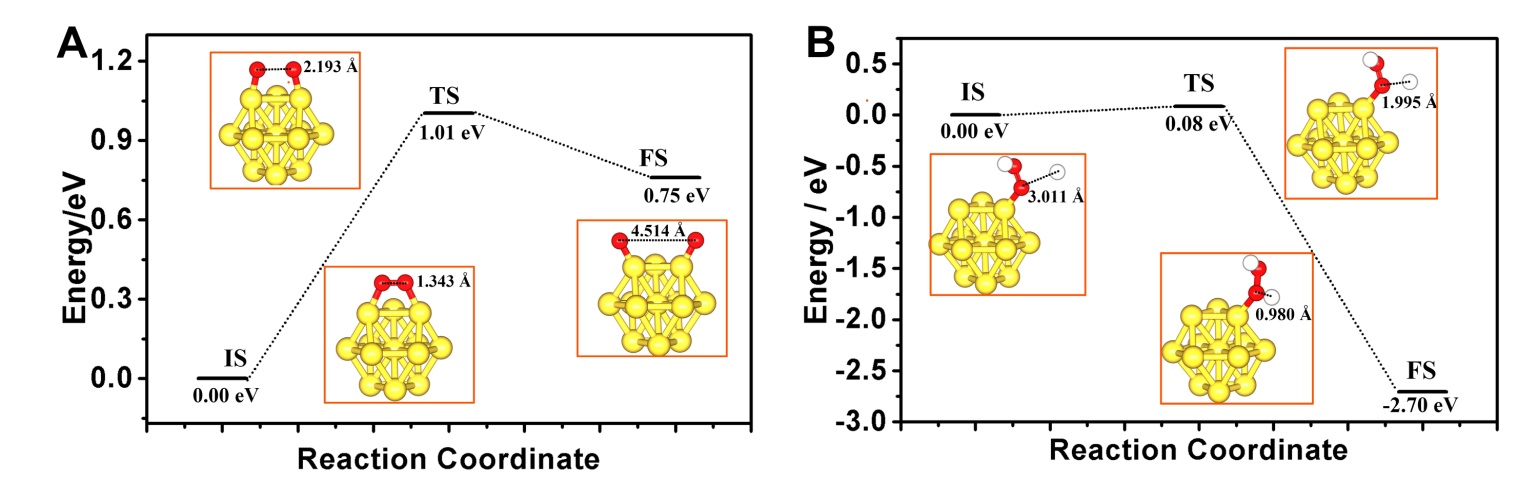


**FIGURE S9**│The structures for initial state (IS), transition state (TS), final state (FS) and potential energy profile for **(A)** *O_2_→ *O + *O, **(B)** *OOH + H^+^ + e^-^ → H_2_O_2_* on Au_13_ cluster.


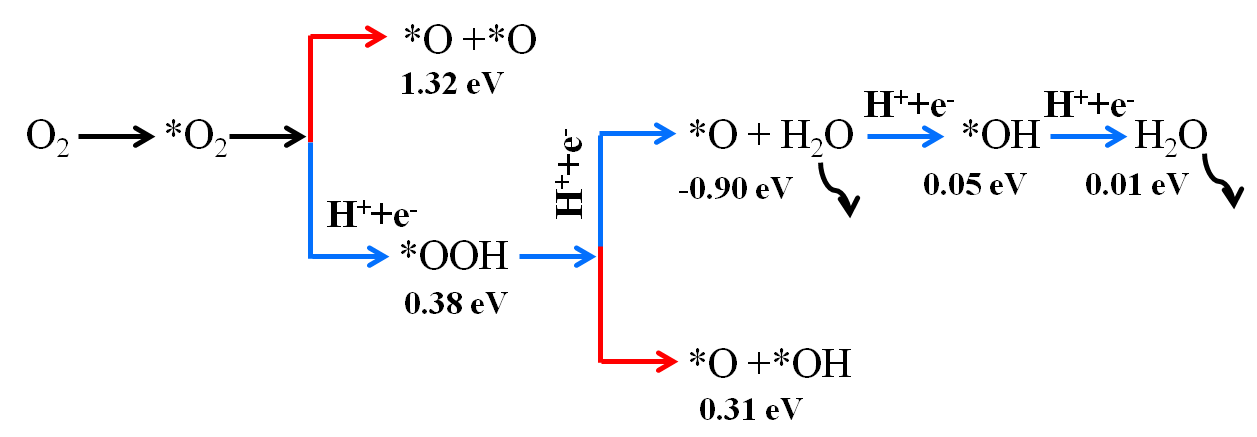


**FIGURE S10**│The possible reaction pathways for ORR on Au/p-BN-V_N_ surface. The * denotes the adsorption site on the Au/p-BN-V_N_ surface. The values (in units of eV) represent the activation energy. The paths with the blue sign is the most favorable due to their lower activation energies.

**2 Supplementary Tables**

**Table S1**│ The adsorption energies (*E*_ads_, eV), O-O lengths, and charges transfer amount from catalysts to O_2_ obtained by the Hirshfeld Charge analysis of the adsorbed *O_2_-Bridge on metal (Pt, Pd, and Au)/p-BN with B and N vacancy defect.

| Catalysts | *E*_ads_ O_2_ (eV) | *d*_O-O_ (Å) | *d*_M-O_ (Å) | Charge (e) |
| --- | --- | --- | --- | --- |
| Pt/p-BN-V_B_ | 1.043 | 1.312 | 2.016 | 0.202 |
| Pt/p-BN-V_N_ | 2.213 | 1.347 | 2.141 | 0.230 |
| Pd/p-BN-V_B_ | 0.611 | 1.302 | 2.070 | 0.205 |
| Pd/p-BN-V_N_ | 1.976 | 1.336 | 2.070 | 0.343 |
| Au/p-BN-V_B_ | 0.522 | 1.280 | 2.008 | 0.128 |
| Au/p-BN-V_N_ | 1.658 | 1.489 | 1.979 | 0.513 |

**Table. S2**│ The calculated adsorption energies (*E*_ads_/eV) of *O_2_, *OOH, *O, *OH and H_2_O on Au/p-BN-V_N_, which obtained in a H_2_O solvent environment by COSMO.

|  | O_2_ (Bridge) | O_2_(Pauling) | OOH | OH | O | H_2_O |
| --- | --- | --- | --- | --- | --- | --- |
| E_ads_/eV | 1.658 | 0.717 | 1.925 | 3.282 | 5.413 | 0.488 |
| E_ads_/eV(COSOM) | 2.031 | 1.150 | 1.911 | 3.274 | 5.752 | 0.509 |
